# A new treatment strategy for Kienböck’s disease: combination of bone marrow transfusion, low-intensity pulsed ultrasound therapy, and external fixation

**DOI:** 10.1007/s00776-012-0332-7

**Published:** 2012-11-01

**Authors:** Takeshi Ogawa, Naoyuki Ochiai, Yasumasa Nishiura, Toshikazu Tanaka, Yuki Hara

**Affiliations:** 1 Department of Orthopaedic Surgery, Kikkoman General Hospital, 100 Miyazaki, Noda, Chiba 278-0005 Japan; 2 Department of Orthopaedic Surgery, Institute of Clinical Medicine, Graduate School of Comprehensive Human Sciences and University Hospital, University of Tsukuba, 1-1-1 Tennodai, Tsukuba, Ibaraki 305-8575 Japan; 3 Tsuchiura Clinical Education and Training Station, Tsukuba University Hospital, 2-7-14 Shimotakatsu, Tsuchiura, Ibaraki 300-8585 Japan

## Abstract

**Background:**

The purpose of this study was to investigate the midterm clinical and radiographic outcomes of this new treatment for Kienböck’s disease.

**Methods:**

We applied a new method involving drilling, bone marrow transfusion, external fixation, and low-intensity pulsed ultrasound for patients with Kienböck’s disease. Between 2000 and 2006, the treatment was performed in 18 patients (10 men and 8 women; 9 right wrists and 9 left wrists). The preoperative Lichtman stages were stage II in 4 cases, stage IIIa in 11 cases, and stage IIIb in 3 cases. The mean age at surgery was 44.9 years (range 16–68 years), and the mean follow-up period was 63 months (range 28–125 months). The overall results were evaluated using the Mayo wrist score and Nakamura scoring system for Kienböck’s disease. Magnetic resonance imaging (MRI) was performed for all patients.

**Results:**

Wrist pain improved to no pain in 13 patients, mild pain in 4 patients, and moderate pain in 1 patient. The average wrist flexion–extension arc was 100° and averaged 120 % of the preoperative value. The average grip strength increased from 50 to 85 % relative to the unaffected side. On roentgenograms, the carpal height ratio (change from 0.53 to 0.51) and the Stahl index (change from 0.38 to 0.32) decreased slightly. On MRI, fatty marrow was recovered in 11 patients (61 %) on proton density-weighted images.

**Conclusions:**

This method can be used as a less-invasive surgical treatment alternative for Kienböck’s disease. At an average follow-up period of 6 years, this new treatment has been shown to be a reliable and durable procedure for patients with Lichtman stage II or stage III Kienböck’s disease. Caution should be exercised for patients with a fragmented lunate because of the risk of collapse and nonunion of the lunate.

## Introduction

Kienböck’s disease is a progressive wrist disorder characterized by osteonecrosis of the lunate. The treatment of Kienböck’s disease remains controversial, although numerous surgical procedures have been described [1–10]. These surgical methods have some disadvantages, such as large, invasive, and complicated procedures and uncertain lunate bone regeneration. We considered that a less invasive method would improve the clinical symptoms and lead to regeneration of the lunate bone itself. We developed a new treatment for Kienböck’s disease in 2000. At that time, bone marrow (BM) transfusion had been applied for osteosynthesis, delayed union, and bone defects [11, 12]. Osteogenic precursor cells, which are capable of producing bone, have been demonstrated to be present among the stromal and endothelial cells of the BM [13]. It has also been shown that endothelial progenitor cells, CD34-positive cells, have vasculogenic properties and originate from the BM [14]. Low-intensity pulsed ultrasound (LIPUS) has been adopted for delayed union since successful experiments were first reported by Duarte [15]. In addition, application of LIPUS has been shown to accelerate the healing of fresh tibial fractures and distal radial fractures [16, 17]. On the basis of these findings, we applied a new method involving drilling, BM transfusion, external fixation (EF), and LIPUS to 4 patients with Lichtman stage II or III Kienböck’s disease. We obtained good clinical results with a three-year follow-up. To better understand the treatment described in this study, we histopathologically examined the efficacies of drilling, BM transfusion, and LIPUS for regeneration of necrotic bone in a rabbit small-bone model. BM injection with drilling was effective for revitalizing a severely necrotic small bone in the experimental rabbit model [18]. Moreover, we verified that LIPUS therapy accelerated bone regeneration in this model [19]. The purpose of this study was to investigate the midterm clinical and radiographic outcomes for a total of 18 patients with Kienböck’s disease treated by drilling, BM transfusion, and LIPUS.

## Materials and methods

### Subjects

Between 2000 and 2006, the treatment was performed in 18 patients with Kienböck’s disease (10 men and 8 women; 9 right wrists and 9 left wrists) who visited the outpatient department of our hospital. The research committee of the hospital approved this human study. Nine dominant and 9 nondominant sides were affected. At the time of surgery, 3 patients were students and 15 patients were employed (6 construction workers, 3 home workers, 3 agriculture workers, 2 office workers, and 1 truck driver). The preoperative Lichtman stages were stage II in 4 patients, stage IIIa in 11 patients, and stage IIIb in 3 patients. The ulnar variance was neutral in 9 patients, positive in 4 patients, and negative in 5 patients. The mean age at surgery was 44.9 years (range 16–68 years) and the mean follow-up period was 63 months (range 28–125 months). Our indications for surgical intervention were diagnosis of Kienböck’s disease at stages II, IIIa, and IIIb.

### Surgical and treatment protocols

**Fig. 1:**
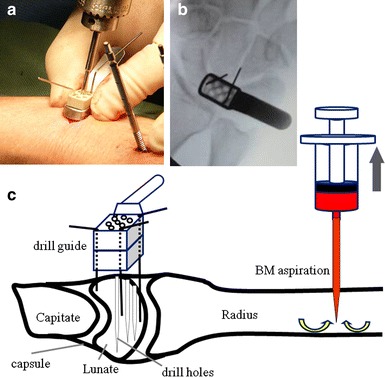
Surgical procedure. **a** Drilling of the lunate with our original drill guide. **b** Radiograph obtained when the drill guide was set. **c** Schema of bone marrow (BM) aspiration

**Fig. 2:**
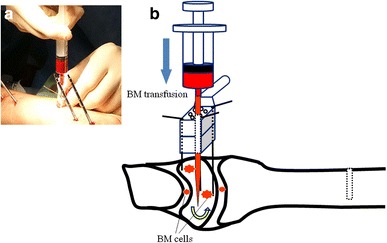
**a** We transfused the bone marrow (BM) into the lunate by direct injection through the radiolucent drill guide based on our profile over the joint capsule. **b** Schema of the BM transfusion

**Fig. 3:**
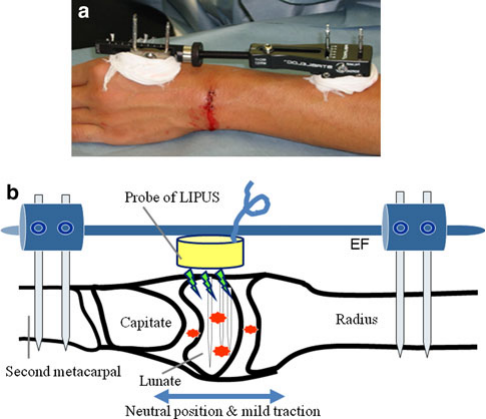
**a** Setting the EF in a neutral position with mild traction of the wrist. **b** Schema of the EF setting and LIPUS stimulation

Under general anesthesia, we inserted two pins into the second metacarpal and radial diaphysis to install a bridging external fixator. We created a 2 cm transverse incision over the lunate, retracted the extensor tendon with preservation of the joint capsule, and placed a radiolucent drill guide based on our profile over the joint capsule. Next, we drilled three holes with a 2 mm diameter drill (Fig. 1a, b). We collected BM samples (approximately 2 mL) from the radius by aspiration using a 10 mL syringe with an 18-gauge needle (Fig. 1c). We transfused the BM into the lunate through the drill holes to fill the space (approximately 2 mL; Fig. 2a, b). The external fixator (Stableloc; Kobayashi Medical, Tokyo, Japan) was fixed in slight traction with the wrist in a neutral position, and was removed after 8 weeks (Fig. 3a, b). We applied traction to the radiocarpal and intercarpal joints to create a 1 mm opening to achieve distraction of the joints. The LIPUS therapy was applied using a Sonic Accelerated Fracture Healing System (SAFHS; Teijin Pharma, Tokyo, Japan). The treatment head module delivered an ultrasound signal composed of a burst width of 200 μs of 1.5 MHz sine waves, with a repetition rate of 1 kHz and an average spatiotemporal intensity of 30 mW/cm^2^. The head module was placed dorsally relative to the wrist, and the skin was protected using a standard ultrasound gel. LIPUS therapy was introduced daily for 20 min, similar to its use to treat bone nonunion or delayed union [15–17]. The period of LIPUS ranged from 4 weeks, which allowed complete wound healing, to 4 months, which allowed recovery of the range of motion to some extent.

### Assessment

Both preoperatively and postoperatively, all patients were interviewed and examined for wrist pain, range of motion, grip strength, and imaging studies by two of the authors (T.O. and Y.H.) who did not perform any of the surgeries. Pain was assessed using four grades [20]: no pain; occasional mild pain that occurred when the wrist was exposed to cold or increased workload; tolerable moderate pain that occurred anytime at rest or at work; and severe to intolerable pain. The overall results were evaluated using the Mayo wrist score [20] and the Nakamura scoring system for Kienböck’s disease (Nakamura score) [3, 6]. At the follow-up evaluation, a roentgenogram was obtained to assess the carpal height ratio (CHR) and the Stahl index (SI). Magnetic resonance imaging (MRI) was performed for all patients within 1 month of surgery and annually thereafter, using a 1.5 T system (Gyroscan NT Intera; Philips Medical Systems, Best, The Netherlands). Coronal two-dimensional proton density weighted (PDW) images and fast field echo (FFE) images of the wrist were acquired using a 47 mm microscopy surface coil (Philips Medical Systems). The slice thickness was 1.5 mm and the slice interval was 0.1 mm with a field of view of 50 mm. Under these conditions, the lunate bones were imaged in eight slices in the coronal view. In general, PDW images of normal lunates exhibit high signal intensities, whereas FFE images exhibit intermediate intensities. In comparison, PDW images of the necrotic lunate demonstrate lower signal intensities, while FFE images exhibit higher or lower intensities [21]. We evaluated all eight slices. We considered that patients showed improvement on MRI when the signals changed to nearly normal on at least four of the eight slices.

### Statistical analysis

We compared the clinical and radiographic results obtained before and after surgery and analyzed the differences using a paired *t* test. Differences were considered to be significant when the *p* value was <0.05.

## Results

Preoperative wrist pain was moderate in 15 patients and severe in 3 patients. At the final follow-up, the wrist pain had improved in all patients, specifically to no pain in 13 patients, mild pain in 4 patients, and moderate pain in 1 patient (Table 1).

**Table 1:** Summary of the clinical results

Case	Sex	Age at operation (years)	Follow-up period (months)	Wrist pain		Grip strength^a^		ROM (°) wrist extension		ROM (°) wrist flexion	
				Pre-op	Post-op	Pre-op	Post-op	Pre-op	Post-op	Pre-op	Post-op
1	M	56	125	Moderate	None	90	85	40	45	50	40
2	F	66	58	Moderate	None	75	105	45	60	30	45
3	F	62	58	Moderate	None	75	135	45	60	30	75
4	F	24	103	Severe	None	130	125	70	65	60	60
5	M	68	84	Severe	None	45	90	20	45	25	45
6	M	44	90	Moderate	None	60	90	30	50	30	40
7	F	65	87	Moderate	None	65	85	30	45	35	40
8	F	19	21	Moderate	Mild	70	100	40	60	30	40
9	M	27	49	Severe	None	60	110	45	60	15	50
10	F	50	84	Severe	None	80	95	45	50	35	45
11	M	26	41	Moderate	Mild	70	75	40	35	30	40
12	F	60	72	Severe	Moderate	80	100	50	60	30	40
13	M	16	25	Moderate	None	70	105	40	60	30	45
14	M	26	36	Severe	Mild	80	90	30	30	50	60
15	M	41	68	Severe	None	110	115	60	60	50	55
16	M	53	65	Moderate	None	120	135	65	70	55	65
17	M	22	36	Moderate	None	95	100	45	45	40	55
18	F	61	36	Moderate	Mild	60	105	35	60	25	45
Mean ± SD			62.9 ± 28			77.9 ± 22	102.5 ± 16	43.0 ± 12	53.3 ± 10	36.1 ± 12	49.1 ± 10
*p* value						0.99		0.99		0.99	

^a^The grip strength is given as the percentage of the strength on the unaffected side

The preoperative values of wrist extension and flexion ranged from 20° to 70° (mean 43.0°) and from 15° to 60° (mean 36.1°), respectively. At the final follow-up, the mean wrist extension angle was 53.3° (range 30–70°) and the mean wrist flexion angle was 49.1° (range 40–75°). Comparisons of the preoperative and postoperative values in all patients showed no significant postoperative improvements in wrist extension (*p* = 0.99) and flexion (*p* = 0.99; Table 1).

The preoperative mean grip strength was 77.9 % (range 45–130 %) relative to the unaffected side. At the final follow-up, the mean grip strength was 102.5 % (range 75–135 %) relative to the unaffected side. Comparisons of the preoperative and postoperative values in all patients showed no significant postoperative improvements in grip strength (*p* = 0.99; Table 1).

On radiographs, lunate collapse had progressed in most cases. The CHR decreased significantly from 0.53 to 0.51 (*p* = 0.046). The SI decreased significantly from 0.38 to 0.32 (*p* = 0.001). In all cases of progressive collapse, these radiographic changes occurred within one year after removing the EF. On MRI, fatty marrow was recovered in 11 patients (61 %) on PDW images (Table 2). This improvement on MRI emerged over the course of 1–2 years postoperatively.

**Table 2:** Summary of the radiologic results

Case	Lichtman’s stage		CHR		Stahl index		Nakamura’s score	Mayo wrist score	MRI
	Pre-op	Post-op	Pre-op	Post-op	Pre-op	Post-op			
1	IIIb	IIIb	0.46	0.51	0.20	0.22	Good	Good	I
2	II	IIIa	0.56	0.55	0.43	0.44	Excellent	Good	I
3	II	IIIa	0.56	0.50	0.43	0.33	Good	Excellent	I
4	IIIa	IIIa	0.50	0.51	0.41	0.40	Good	Excellent	I
5	IIIa	IIIa	0.58	0.53	0.47	0.33	Good	Fair	U
6	IIIa	IIIa	0.54	0.54	0.45	0.26	Excellent	Excellent	I
7	II	IIIb	0.55	0.55	0.36	0.28	Good	Excellent	U
8	IIIa	IIIb	0.45	0.47	0.35	0.27	Good	Good	I
9	IIIa	IIIa	0.60	0.48	0.33	0.24	Good	Fair	U
10	IIIa	IIIa	0.53	0.46	0.40	0.42	Good	Excellent	I
11	IIIa	IIIa	0.59	0.48	0.40	0.45	Fair	Poor	U
12	IIIa	IIIa	0.53	0.53	0.40	0.32	Fair	Poor	U
13	IIIa	IIIa	0.49	0.47	0.37	0.33	Good	Fair	I
14	IIIb	IIIb	0.48	0.46	0.31	0.28	Fair	Fair	U
15	II	IIIa	0.57	0.60	0.47	0.42	Good	Excellent	I
16	IIIa	IIIa	0.64	0.63	0.42	0.27	Excellent	Excellent	I
17	IIIa	IIIa	0.51	0.56	0.33	0.26	Excellent	Excellent	I
18	IIIb	IIIb	0.49	0.47	0.35	0.35	Good	Good	U
Mean (and SD)			0.53 ± 0.05	0.51 ± 0.04	0.38 ± 0.06	0.32 ± 0.07			
*p* values			0.046		0.001				

*I* improved, *U* unchanged

**Fig. 4:**
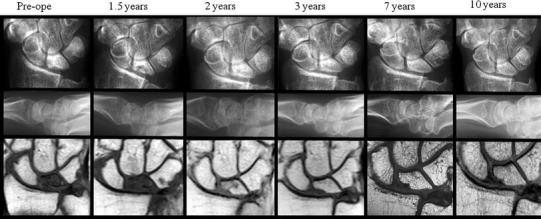
Roentgenograms and MRI for case 4. The images show that the lunate has not collapsed, and that the lunate intensity is recovered on MRI. The *upper panels* show anteroposterior views on the roentgenograms, the *middle panels* show lateral views on the roentgenograms, and the *lower panels* show PDW images on MRI. The preoperative images are shown on the *left*, together with the 1.5-, 2-, 3-, 7-, and 10-year postoperative images

At the final follow-up evaluation, the Mayo wrist score was excellent in 8 patients, good in 4 patients, fair in 4 patients, and poor in 2 patients, while the Nakamura score was excellent in 4 patients, good in 11 patients, fair in 3 patients, and poor in no patients (Tables 1, 2). The roentgenogram and MRI findings of a representative case (case 4) are shown in Fig. 4. The CHR changed from 0.50 to 0.51 and the SI changed from 0.41 to 0.40. The lunate intensity on MRI showed improvement at 1.5 years postoperatively, and this was maintained even at 10 years postoperatively. The Nakamura score was good and the Mayo wrist score was excellent.

## Discussion

The following treatments for Kienböck’s disease have been reported to have excellent outcomes: conservative therapy [2], radial osteotomy [3, 6, 8], cancellous bone grafting with EF [4], metaphyseal core decompression [5], vascularized bone grafts [7], proximal row carpectomy [9], and capitate shortening osteotomy [10]. However, the outcomes of our method were never inferior to those of the abovementioned conventional treatments (Table 3). When comparing the different procedures, the most noticeable finding in our clinical results was the improvement in grip strength. With respect to pain, this improved with almost any treatment. On the other hand, radiographs showed significant lunate collapse. For radial shortening osteotomy, Koh et al. [6] reported that the CHR changed from 0.52 to 0.51 and the SI changed from 0.31 to 0.28, while Watanabe et al. [8] reported that the CHR changed from 0.52 to 0.51 and the SI changed from 0.43 to 0.40. Aspenberg et al. [22] reported that the lunate harvested at one year after creation in a monkey lunate malacia model was radiographically flattened, although the joint cartilage was still normal in this model. Most of the subchondral bone shell was replaced with newly vascularized bone. They speculated that revascularization was the probable cause of the collapse. In our previously reported animal experiments, we observed that BM-transfused necrotic bone maintained its original shape for at least 8 weeks, although the original shape was lost by 12 weeks [18]. It is likely that the necrotic bone started to lose its integrity at 8 weeks during the regenerative process, and this may have been the result of high bone turnover, which was predominantly facilitated by bone resorption. At 20 weeks, the collapsed bones in our previous study were not soft and showed elasticity [18]. Considering these results, slight collapse of the lunate with progression was inevitable for revitalization of the necrotic lunate. In our treatment, EF may reduce mechanical stress on the lunate, and it was used in an attempt to maintain the CHR and SI. In this study, we maintained traction for only 8 weeks to minimize the risk of infection or contracture. However, extending the EF period to 12 weeks or more might be beneficial.

**Table 3:** Comparison of clinical and radiological results obtained with different procedures

Source	Procedure	Follow-up period (months)	Wrist pain	Grip strength	ROM (°) wrist extension		ROM (°) wrist flexion		CHR		SI		Nakamura score (E/G^c^ %^a^)	Mayo wrist score (E/G^c^ %^a^)	MRI (improved %^a^)
			None or mild (%^a^)	Post-op (%)^b^	Pre-op	Post-op	Pre-op	Post-op	Pre-op (mean)	Post-op (mean)	Pre-op (mean)	Post-op (mean)			
Kristensen et al. [2]	Conservative	246	80 (39/49)	–	–	–	–	–	–	Deformed	–	Deformed	–	–	–
Koh et al. [6]	Radial osteotomy	125	96 (24/25)	85	67 % (pre-op)^b^; 82 % (post-op)^b^				0.52	0.51	0.31	0.28	68 (15/25)	96 (24/25)	–
Croog and Stern [9]	Proximal row carpectomy	120	88 (16/18)	88	–	56	–	49	Radiocapitate OA 87 %			–	–	67 (12/18)	–
Watanabe et al. [8]	Radial osteotomy	115	100 (13/13)	73	46	58	40	54	0.52	0.51	0.43	0.40	–	50 (6/12)	–
Illarramendi et al. [5]	Metaphyseal core decompression	115	91 (20/22)	75	–	59	–	57	–	–	–	–	–	–	80 (4/5)
Zelouf and Ruby [4]	EF + cancellous bone grafting	56	82 (14/17)	81	53	54	52	59	0.50	0.49	–	–	–	71 (12/17)	50 (5/10)
Moran et al. [7]	Vascularized bone graft	31	92 (24/26)	89	–	50	–	44	–	0.46	–	–	–	46 (12/26)	70 (12/17)
Afshar [10]	Capitate shortening osteotomy	12	100 (9/9)	Improved	–	Improved	–	Improved	–	–	–	–	–	–	100 (9/9)
This study	BMT + LIPUS + EF	63	94 (17/18)	102	43	53	36	49	0.53	0.51	0.38	0.32	83 (15/18)	67 (12/18)	61 (11/18)

^a^Improved cases/total cases

^b^Percentage of the strength on the unaffected side

^c^Excellent or good

Regarding the number of cells used for transfusion, there is a report that healthy human adult BM osteoprogenitors represent approximately 0.001–0.01 % of the nucleated cells, according to a homogeneous population of human mesenchymal cells isolated from BM taken from the iliac crest [23]. However, there are no reports of the number of mesenchymal stem cells in the BM of the radius, and we were unable to determine the proportion of mesenchymal stem cells in the radius. BM transplantation as a treatment for osteonecrosis of the femoral head has been reported to be more effective when a larger number of cells is transplanted [24]. On the other hand, Connolly [25] described that concentrated BM is not always necessary for a small bone, e.g., the carpal scaphoid. We also supposed that the whole BM contained endothelial progenitor cells, platelets, cytokines, and growth factors [26]. Asahara et al. [14] reported that postnatal neovascularization does not rely exclusively on sprouting from preexisting blood vessels (angiogenesis); instead, endothelial progenitor cells circulate from the BM to become incorporated into and thus contribute to postnatal physiological and pathological neovascularization, which is consistent with postnatal vasculogenesis. Based on these findings, the BM transfusion that we performed is similar to the injection of osteoprogenitor cells, and vasculogenesis can also be expected. We are sure that direct transfusion of whole BM makes sense. The effects of LIPUS for Kienböck’s disease are unclear. In previous studies, LIPUS stimulation directly affected osteogenic cells, leading to mineralized nodule formation [27]. LIPUS therapy may provide an alternative noninvasive method for osteoblast and tissue regeneration to enhance osteogenesis. Takayama et al. [27] reported that LIPUS stimulation did not affect the rate of cell proliferation but instead increased osteogenic differentiation. In addition, LIPUS can improve the blood flow. Barzelai et al. [28] reported the effects of LIPUS on tissue blood flow and angiogenesis after limb ischemia in vivo. Rawool et al. [29] described that power Doppler sonography revealed increased vascularity around the fracture sites in LIPUS-treated dogs. In our animal study [19], LIPUS alone and LIPUS plus bone drilling were not effective for revitalizing severely necrotic small tarsal bones in rabbits. However, the combined method involving drilling, BM injection, and LIPUS for small-bone necrosis significantly accelerated new bone formation at 8 weeks [19]. Therefore, it is considered that LIPUS in conjunction with BM injection synergistically promotes angiogenesis, vasculogenesis, and bone formation. Kienböck’s disease is denoted an avascular necrosis because blood vessels are usually absent. In the lunates in our other study, the trabecular bone structures were segmented and fatty marrow was absent, which potentially allows the formation of fibrous granulation tissues, albeit in the presence of blood vessels [21]. Moreover, nutrient vessels enter the lunate in a distal-to-proximal direction via the palmar and dorsal horns and build an intraosseous vascular network [30]. If the progenitor cells undergo repeated differentiation and proliferation in the limited space of the lunate (which serves as a scaffold), this can be expected to result in revitalization of the necrotic lunate.

The present study has several limitations. First, the study was retrospective and the number of cases was small. Second, we could not determine the proportion of mesenchymal stem cells in the transfused BM. Third, we have no evidence for the appropriateness of the selected periods of LIPUS (from 4 weeks to 4 months) and EF (8 weeks). Fourth, there were no controls, and the relative contribution of each of treatment modality (BM transplant, LIPUS, and EF) to the overall results cannot be estimated. Finally, caution should be exercised for patients with a fragmented lunate due to the risk of collapse and nonunion of the lunate.

In summary, we applied a new method involving drilling, BM transfusion, EF, and LIPUS to 18 patients with Lichtman stage II or stage III Kienböck’s disease. At the final follow-up, the wrist pain had improved in all patients, but the range of motion and grip strength did not show any significant postoperative improvements. On radiographs, lunate collapse had progressed in most cases. On MRI, fatty marrow was recovered in 11 patients (61 %). Nakamura’s total assessment score was excellent in 4 patients, good in 11 patients, fair in 3 patients, and poor in no patients. At an average follow-up period of six years, this method can be used as a less-invasive surgical treatment, and has been shown to be a reliable and durable procedure for patients with Lichtman stage II or stage III Kienböck’s disease.
